# Continuous ab interno repairing of traumatic cyclodialysis cleft using a 30-gauge needle in severe ocular trauma: a clinical observation

**DOI:** 10.1186/s12886-019-1274-z

**Published:** 2019-12-26

**Authors:** Haibo Li, Jinhong Cai, Xiaofeng Li

**Affiliations:** Department of Ophthalmology, Xiamen Eye Centre of Xiamen University, Xiamen, 361016 Fujian Province China

**Keywords:** Traumatic cyclodialysis cleft, Continuous mattress suture, Ocular trauma

## Abstract

**Background:**

To investigate the efficacy and safety of continuous ab interno repairing of traumatic cyclodialysis cleft in severe ocular trauma using a 30-gauge (G) needle.

**Methods:**

Fifteen patients (15 eyes) with traumatic cyclodialysis cleft admitted to the ocular trauma department of our hospital from July 2014 to December 2018 were included in this study. After the bulbar conjunctiva corresponding to the ciliary body was incised along the corneal limbus, an incision was made along the corneal limbus on the opposite side. A 30G needle with a 10–0 suture entered the anterior chamber from the incision and passed through the ciliary body with clefts and the sclera to fixate the ciliary body on the sclera wall with continuous mattress suture. The best corrected visual acuity (BCVA) and intraocular pressure (IOP) were observed preoperatively and postoperatively. In vivo ultrasound biomicroscopy (UBM) was performed to observe closure of cyclodialysis cleft before and after surgery.

**Results:**

Fifteen patients successfully underwent continuous mattress suture for repair of cyclodialysis cleft. No bleeding and suture breakage were reported during surgery. After surgery, the UBM during follow-up showed satisfactory closure of the cyclodialysis cleft. The BCVA and IOP were improved to different degrees. The difference between the preoperative IOP and the postoperative IOP (1 week) was statistically significant (preoperative: 6.49 ± 0.98 mmHg, postoperative: 16.17 ± 4.65 mmHg, t = − 8.43, *P* < 0.05), and the difference between the preoperative IOP and the postoperative IOP (1 month) was also statistically significant (preoperative: 6.49 ± 0.98 mmHg, postoperative: 14.63 ± 3.63 mmHg, t = − 8.38, *P* < 0.05). Duration of outpatient follow-up was 3 to 12 months. No complications, including exposed knots, loose sutures, decompensation of corneal endothelium, sympathetic ophthalmia, endophthalmitis and choroidal detachment, were reported.

**Conclusion:**

Continuous ab interno repairing of traumatic cyclodialysis cleft in severe ocular trauma using a 30G needle is a safe and effective procedure with simple operation, little tissue damage and few complications.

## Background

Cyclodialysis cleft is a common complication of ocular trauma and is occasionally caused by iatrogenic injury. Small cyclodialysis clefts can be treated with topical application of atropine or laser. Cyclodialysis clefts that are unresponsive to conservative treatments or large cyclodialysis clefts require surgical intervention. Many surgical approaches are available in clinical practice [1]. In this study, we introduce a simple technique, continuous ab interno method, to repair cyclodialysis cleft using a 30-gauge (G) needle in severe ocular trauma. The technique is reported as follows.

## Methods

### I. General data

Patients: Fifteen patients (15 eyes) with traumatic cyclodialysis cleft who were admitted to the Ocular Trauma Department of Xiamen Eye Centre of Xiamen University from July 2014 to December 2018 were included in this study (Table 1). There were 10 males and 5 females, with a mean age of 45.60 ± 12.57 (range: 24 to 65) years. The affected eyes included 8 right eyes and 7 left eyes. The causes of injury included stone (3 cases), wood (3 cases), elbow (2 case), bamboo chip (1 case), cutting blade (1 case), explosion (1 case), table corner (1 case), badminton ball (1 case), water cannon (1 case), and soccer ball (1 case). Occupations included workers (8 cases), farmers (3 cases), and clerks (4 cases). All injuries were mechanical globe injuries, of which 4 were open globe injuries (case 2,3,4,5 in Table 1) and 11 were closed globe injuries. Two of the 4 patients with open globe injuries had primary wound closure in rural hospital; the others had primary wound closure in the urgent care of our hospital. Of the 15 patients, in addition to cyclodialysis cleft, iridodialysis was present in 5 patients, lens subluxation in 13 patients, traumatic cataract in 5 patients, vitreous haemorrhage in 15 patients, and retinal detachment in 5 patients.

**Table 1:** Demographic data of 15 patients undergoing continuous ab interno repairing of cyclodialysis clefts

No.	Sex/ Age ranges	OD/OS	Causes	Extent of clefts by UBM/clock hours	Associated pathology	Surgery	BCVA/log MAR		IOP/mmHg	
							Preoperative	Postoperative	Preoperative	Postoperative/1 week
1	1/40–49	OD	Stone	2.5	Lens subluxation, hyphema, vitreous haemorrhage, iridodialysis, macular oedema	Cyclopexy+PPL + PPV + iridodialysis repair	2.30	1.30	6.5	26.2
2	2/40–49	OD	Bamboo	3	Lens subluxation, hyphema, vitreous haemorrhage, iridodialysis, choroidal detachment	Cyclopexy+PPL + PPV + iridodialysis repair	2.30	0.30	5.7	11.0
3	1/50–59	OD	Firecracker	5	Traumatic cataract, hyphema, iridodialysis, vitreous haemorrhage, RD, choroid detachment	Cyclopexy+PPL + PPV + silicone oil	1.85	1.30	6.8	13.6
4	1/40–49	OS	Grinding wheel	6	Lens subluxation, hyphema, vitreous haemorrhage, RD, choroid detachment	Cyclopexy+PPL + PPV + silicone oil	2.60	1.60	6.7	19.4
5	2/50–59	OS	Wood	5	Lens subluxation, hyphema, vitreous haemorrhage, iridodialysis, choroidal detachment	Cyclopexy+PPL + PPV + C2F6	1.85	0.70	6.6	9.0
6	2/40–49	OD	Table	5	Lens subluxation, hyphema, vitreous haemorrhage, mydriasis, choroidal detachment	Cyclopexy+PPL + PPV	2.30	0.40	8.7	18.8
7	1/20–29	OD	Badminton	4	Lens subluxation, hyphema, mydriasis, choroidal detachment, vitreous haemorrhage	Cyclopexy+PPL + PPV	2.30	0.40	5.4	15.4
8	1/60–69	OS	Stone	3.5	Traumatic cataract, vitreous haemorrhage, RD, choroid detachment	Cyclopexy+PPL + PPV + silicone oil	2.30	1.30	7.3	16.2
9	1/60–69	OS	Wood	4	Lens subluxation, traumatic cataract, hyphema, vitreous haemorrhage, macular oedema, choroid detachment	Cyclopexy+phaco+PPV	2.30	0.50	5.0	11.2
10	1/20–29	OD	Football	4	Lens subluxation, hyphema, mydriasis, vitreous haemorrhage	Cyclopexy+PPL + PPV	1.85	0.50	6.0	18.2
11	2/30–39	OS	Elbow	3	Lens subluxation, traumatic cataract, hyphema, RD, choroid detachment, vitreous haemorrhage	Cyclopexy+phaco+PPV + silicone oil	1.85	1.30	7.6	22.6
12	2/60–69	OD	Stone	4	Hyphema, lens subluxation, vitreous haemorrhage, choroid detachment	Cyclopexy+PPL + PPV	1.85	0.70	5.2	16.4
13	1/20–29	OS	Elbow	3	Hyphema, lens subluxation, vitreous haemorrhage, choroid detachment	Cyclopexy+PPL + PPV	1.85	0.9	6.2	18.0
14	1/50–59	OD	Wood	4	Lens subluxation, traumatic cataract, hyphema, vitreous haemorrhage, macular oedema, RD	Cyclopexy+PPL + PPV + silicone oil	2.3	1.3	6.4	15.4
15	1/40–49	OS	Water cannon	6	Hyphema, lens subluxation, iridodialysis, vitreous haemorrhage, choroid detachment	Cyclopexy+PPL + PPV	1.85	0.1	7.2	11.2

*RD* retinal detachment; phaco: phacoemusification; *PPL* pars plana lensectomy; *PPV* pars plana vitrectomy

### II. Study methods

#### Examination equipment and surgical instruments

Examination equipment and surgical instruments included ultrasound biomicroscopy (UBM) (UBM SW-3200 L panoramic ultrasound biomicroscope, Tianjin Suowei Electronic Technology Co., Ltd.), viscoelastic agent (DisCoVisc, Alcon), surgical suture (10–0 nylon or prolene suture, Johnson & Johnson Inc.), disposable ophthalmic scalpel (15° keratoplasty blade, Alcon), 30G needle (Zhejiang Kangdelai Medical Devices Co., Ltd.), and vitrectomy devices (Bausch & Lomb company).

#### Diagnosis of cyclodialysis cleft and recording method

For the patients with open globe injury, UBM examination was performed 1 week after wound closure. For the patients with closed globe injury, UBM examination was performed when the conjunctival tissue condition allowed. Before the examination, all patients underwent topical anaesthesia with procarbazine 3 times. After immersion of the probe in saline solution, UBM (probe frequency: 50 MHz) examination was performed on the eyeball clockwise from the 12 o’clock position. Attention was paid to the relationships between the ciliary body and the sclera, the angle of the anterior chamber, and the presence of fluid sonolucent area. If the upper end of the ciliary body and the scleral spur were completely separated, the location was recorded as the cyclodialysis cleft site. The examination was performed according to the o’clock position with half o’clock intervals, and all detected sites were recorded. Finally, the condition of the whole ciliary body was assessed according to the records.

#### Surgical procedure

Routine examinations before surgery included slit lamp microscope, corneal endothelial cell count, intraocular pressure (IOP), visual acuity (VA), B-ultrasound, UBM, and fundus examination. For patients with cyclodialysis cleft, atropine was applied locally. Low IOP after conservative treatment and non-healed cyclodialysis cleft detected by UBM were indications for continuous ab interno repairing.

**Fig. 1:**
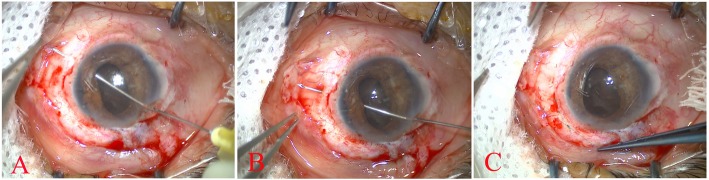
Surgical procedure for continuous ab interno repairing of cyclodialysis cleft using a 30G needle. **a**: The bulbar conjunctiva corresponding to the ciliary body with clefts was incised along the corneal limbus. An incision was made along the corneal limbus on the opposite side, and viscoelastic agent was injected into the anterior chamber through the corneal limbal incision. A 30G needle with a 10–0 suture entered the anterior chamber via the corneal incision opposite to the site of the cyclodialysis cleft, passed through the ciliary body, and exited from the site on the scleral surface 2 mm posterior to the corneal limbus. The tip of the 10–0 suture was pulled and held. **b**: The needle tip was inserted back into the vitreous cavity, passed through the ciliary body, and then exited the sclera again with half o’clock interval. Now, the 10–0 suture formed a loop that was pulled out and locked with the 10–0 suture of the first stich. The needle tip was inserted back into the vitreous cavity. **c**: At completion of suture of all cleft sites, the needle was removed, and the locking-suture loop was adjusted and tied to make the ciliary body attach to the scleral wall


The surgery was performed by a posterior segment surgeon (Haibo Li). For the 15 adult patients in this study, 2% lidocaine combined with 0.75% bupivacaine was used for peribulbar anaesthesia. Based on the location of the cyclodialysis cleft, the bulbar conjunctiva was incised circularly along the corneal limbus, and the sclera vessels were cauterized to stop bleeding. An incision for a 20G needle on the sclera was made at the site 3.5 mm to the lower corneal limbus (temporal side). A 20G 6-mm long perfusion tube was placed and confirmed to be located in the vitreous cavity, and then, the perfusion started. The 23G puncture cannula was placed at the 2 and 10 o’clock positions 3.5 mm posterior to the limbus. The vitrectomy probe was used to remove the opaque lens cortex and nucleus from the back. The hard lens nucleus was removed by the phacoemulsification via an anterior approach (case 9 and 11). A 30G needle with a 10–0 suture entered the anterior chamber via the corneal incision opposite the cyclodialysis cleft site, passed through the starting point of the cyclodialysis cleft, and exited the site on the scleral surface 2 mm posterior to the corneal limbus. The tip of the 10–0 suture was pulled and held. The needle tip was placed back in the vitreous cavity and then passed through the ciliary body and out the sclera again with half o’clock interval. Then, the 10–0 suture formed a loop that was pulled out, and 1 end of the suture passed the loop. The needle tip was inserted back into the vitreous cavity to continue suturing until the cyclodialysis cleft site was reached. The locking-suture loop was adjusted and tied, with the knot buried under the tunica conjunctiva (see the supplementary video in Additional file 1). When the needle was repeatedly moved in and out of the scleral wall, the site of the needle out was kept 2 to 2.5 mm from the limbus (Fig. 1). After the cyclodialysis cleft was closed with the suture, routine pars plana vitrectomy (PPV) was performed. According to the conditions of the retinal tissue of the fundus, membrane stripping, electrocoagulation, intraocular photocoagulation, gas-liquid exchange, and silicone oil filling were performed if necessary.

**Additional file 1.** The surgery video. (AVI 70147 kb)


All procedures were approved by the hospital ethics committee, and informed consent forms were signed by the patients.

### III. Postoperative management and follow-ups

Tobramycin dexamethasone eye drops (4 to 6 times a day) and pranoprofen eye drops (4 times a day) were applied to all patients for 2 weeks, and application was reduced to 2 times a day for an additional 2 weeks. IOP was monitored daily during hospitalization. Local antiglaucoma eyedrops may have been applied if higher IOP was observed. All the patients were followed up for 3 to 12 months, and the VA and IOP of the patients at different time points were recorded. The best corrected visual acuity (BCVA) and IOP before and after surgery were compared. UBM was performed to assess closure of the cyclodialysis cleft after surgery. A slit lamp microscope was used to evaluate the cornea, anterior chamber, retina, loose suture, exposed knots, and other related complications.

### IV. Statistical analysis

SPSS19.0 software was used for statistical analysis. Paired t-test was used to compare the differences in the measurements before and after surgery. *P* < 0.05 was considered statistically significant.

## Results

Fifteen patients successfully underwent continuous ab repairing of cyclodialysis cleft. During surgery, lens removal and vitrectomy were performed concurrently. In 2 patients with hard lens nucleus, phacoemulsification was performed to remove the cataract via the limbus incision. The remaining 13 patients underwent pars plana lensectomy (PPL) to remove the opaque or dislocated lens. Silicone oil endotamponade was used in 5 patients due to rhegmatogenous retinal detachment, and C3F8 gas endotamponade was used in 1 patient. Two patients underwent repair of iridodialysis, using the same continuous mattress suture way.

### I. Range of cyclodialysis cleft

UBM examination was performed 1 week after wound closure in the open globe injury (case 2,3,4,5). For the patients with closed globe injury, UBM examination was performed within the 3 days after trauma. Preoperative UBM examination showed that the range of the clefts was from the 2.5 to 6 o’clock positions, with an average of 4.13 ± 1.08 o’clock positions. The ranges of the clefts were within 1 quadrant (3 o’clock positions) in 4 patients and within 1 to 2 quadrants (3 to 6 o’clock positions) in 11 patients.

### II. BCVA

The mean logMAR BCVA was 2.11 ± 0.26 (range: 1.85–2.60) before surgery and 0.84 ± 0.47 (range: 0.10–1.60) 1 month after surgery. The BCVA was significantly higher after surgery than before surgery (t = 10.13, *P* < 0.05).

### III. IOP

The IOP before and after surgery was measured by non-contact tonometer. The mean IOP was 6.48 ± 0.98 mmHg (range: 5.0–8.7 mmHg, 1 mmHg = 0.133 kPa) before surgery and was 16.17 ± 4.65 mmHg (range: 9.0–26.2 mmHg) at 1 week after surgery. The mean IOP was significantly higher 1 week after surgery than before surgery (t = − 8.43, *P* < 0.05). The mean IOP was 14.63 ± 3.63 mmHg (range: 7.8–22.0 mmHg) 1 month after surgery and was significantly higher than before surgery (t = − 8.38, *P* < 0.05).

During hospitalization after PPV surgery, 5 patients experienced transient high IOP, which subsided after local treatment for lowering IOP. After 2 to 3 weeks, the IOP-lowering medications were discontinued, and the IOP was maintained between 10 and 21 mmHg. In 2 patients, the IOP was still lower than 10 mmHg. The UBM re-examination did not detect any clefts. These results may suggest that impairment of the ciliary body due to severe trauma affects secretion of the aqueous humour of the ciliary body. The filled silicone oil was not able to be removed in these two case, but eyeball atrophy was not reported during the follow-ups.

### IV. Intraoperative and postoperative complications

After continuous mattress suture for repair of traumatic cyclodialysis cleft using a 30G needle, no complications, including detachment of the uvea, iatrogenic iridodialysis, suprachoroidal haemorrhage, anterior chamber haemorrhage, and suture breakage, were reported. Outpatient follow-up was performed for 3 to 12 months. No complications, including exposed knots, loose suture, corneal endothelium decompensation, sympathetic ophthalmia, endophthalmitis, and choroidal detachment, were reported. Three patients experienced tractional retinal detachment due to proliferative vitreoretinopathy and underwent secondary surgery for retinal reattachment. Three months after surgery, 7 patients had improvement of the corrected VA and received intraocular lens implantation. The UCVA was further improved to different degrees.

## Discussion

The cyclodialysis cleft is a separation of the longitudinal muscle of the ciliary body from the scleral spur, creating a direct connection between the anterior chamber and the suprachoroidal space and leading to increase of aqueous outflow, which can cause chronic hypotony, shallow anterior chamber, choroidal detachment, optic disc oedema, retinal folds, and decreased VA. Persistent hypotony may even result in eyeball atrophy [2]. Cyclodialysis cleft is the most common complication in closed globe injuries, with an incidence of 1 to 11% [3]; it occasionally occurs in open globe injuries and iatrogenic injuries [4–6]. In this study, 15 eye injuries were mechanical eye injuries, of which 4 were open globe injuries and 11 were closed globe injuries. In addition to ciliary body damage, tissue damage in the lens, vitreous, and retina were concurrently present in the 15 patients.

**Fig. 2:**
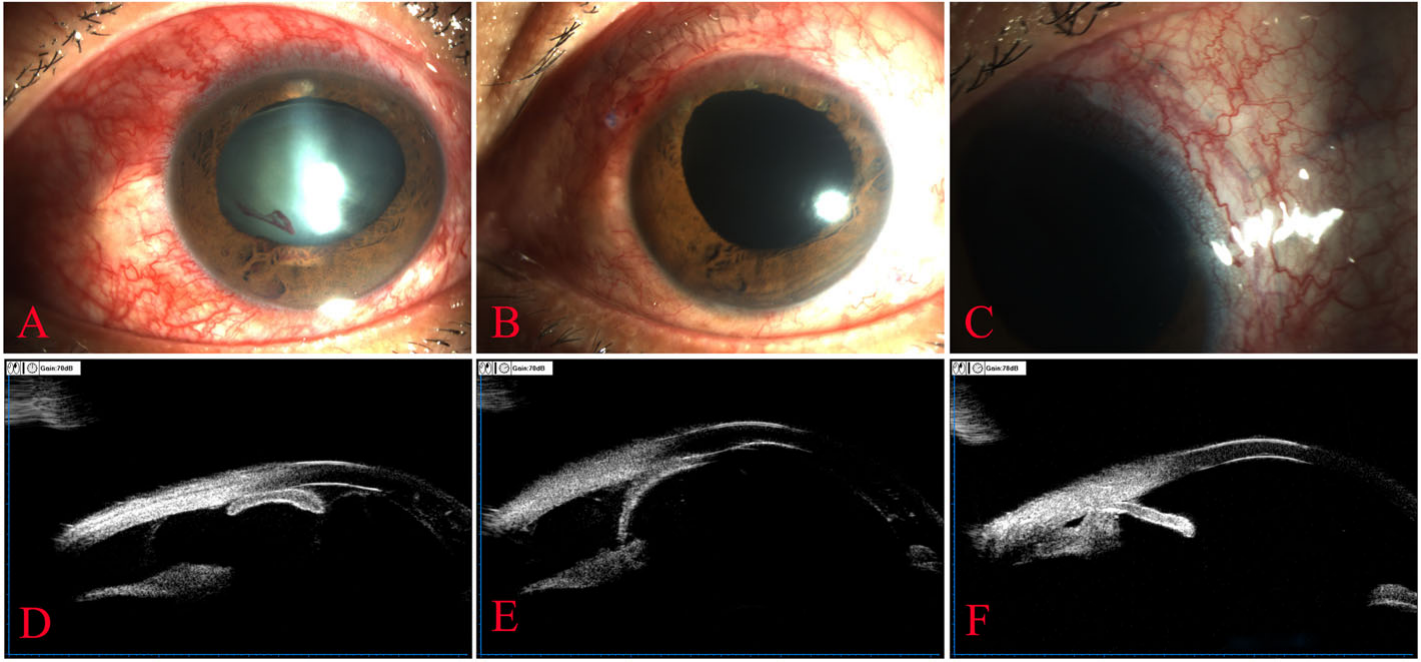
Clinic data of patient 15 undergoing continuous ab interno repairing of cyclodialysis clefs. **b**: Slit lamp examination in patient 15 with closed globe injury. It shows an anterior chamber shallowing with dislocated, cloudy lens, vitreous prolapse, vitreous haemorrhage and choroid detachment. **b**: Patient 15 after continuous ab repairing of the clefts using a 30G needle, PPL and PPV. It shows anterior chamber deepening, aphakia, and stable fundus. **c**: The nylon suture is still visible under the bulbar conjunctiva from 10 o’clock to 4 o’clock. **d**: UBM examination shows complete separation of the ciliary body from the sclera, dislocated lens and iridodialysis at the 12 o’clock position before surgery. **e**: UBM examination shows large cyclodialysis cleft and closed anterior chamber at 2 o’clock position before surgery. **f**: UBM examination at 1 month after continuous ab interno repairing showing that the ciliary body is properly attached to the scleral wall at the 2 o’clock position, and the echo points of the knot behind the sclera

To date, lots of methods are available for diagnosis of cyclodialysis cleft. The preoperative diagnostic methods include gonioscopy, UBM [7, 8], anterior segment optical coherence tomography (AS-OCT) [9], and magnetic resonance imaging (MRI) [10] . The intraoperative diagnostic methods include scleral transillumination [11], anterior chamber perfusion [12], and combined probe exploration [13]. UBM can identify extremely small clefts in high resolution images and is most widely used clinically. In the 15 patients, UBM was performed repeatedly before surgery to determine the exact location of the clefts. Follow-up UBM was also performed to clearly display the conditions of the ciliary body (Fig. 2).

A variety of treatment options are available for cyclodialysis clefts, depending on its size and range. For narrow and small cyclodialysis clefts, relatively conservative treatment can be used, including local atropine [14], argon laser [15], transscleral Nd:YAG laser, and transscleral diode laser therapies [16]. For large cyclodialysis clefts or failure of conservative treatment, surgical intervention is required. Surgical methods include direct or indirect cyclopexy via sclera incision [17, 18], sulcus-fixated Cionni ring and posterior chamber intraocular lens (PCIOL) implantation [19], and vitrectomy combined with gas or silicone oil endotamponade [20]. The success rate varies differently. In our study, conservative atropine therapy was ineffective in all 15 patients before surgery, and repeated UBM showed no sign of clefts healing and consistent low IOP. These findings indicated large clefts that are difficult to self-heal. Ultimately, surgical repair and closure were performed.

Clinically, the direct suture method is more common. After the lamellar scleral flap is created first, the sclera is incised under direct visualization to expose the ciliary body. A 10–0 nylon interrupted suture is used to close the clefts. This method is widely used with high success rates in clinical practice. In 1991, Ormeod et al. reported 28 successful repairs in 29 patients with cyclodialysis cleft [21]. In 2013, Agrawal et al. reported a success rate of 94% [22]. However, this is a complicated method, and highly skilled and experienced surgeons are required to complete the procedure. The surgical procedure requires incision of the sclera near the cleft and exposure of the ciliary body. The suture needle repeatedly enters and exits the ciliary body tissue, which may cause some potential complications, such as intraoperative bleeding, endophthalmitis, cataract, vitreous loss, retinal detachment, scleral incision tear, anterior segment ischaemia, and secondary glaucoma [2, 23]. It is more likely to have long-term issues, such as large scleral scar, thin scleral wall, and staphyloma.

The continuous mattress suture is a common method for iridodialysis repair, in which the needle passes through the tissue medial-laterally with locking stitch to avoid incising the whole layer of the sclera [24]. In 2017, we reported the use of a 30G needle for continuous mattress suture to repair iridodialysis in 19 patients, and the iridodialysis was successfully reinstated by one needle operation. This method was minimally invasive and simple, with few complications [25]. In 2019, Gupta et al. used a 26G needle with a single suture and a single knot to repair large cyclodialysis [26] and suggested it was a simpler and more effective method than other methods. However, a 26G needle is relatively large and may cause pigment membrane detachment, aqueous humour loss, and bleeding when penetrating the scleral wall. To that end, we explored whether the use of a 30G needle for continuous ab interno repairing cyclodialysis cleft can reduce these risks. The 30G needle is often used for vitreous cavity injection in ophthalmology clinics. The outer diameter of the needle is 0.30 mm, and the inner diameter is 0.16 mm. Compared with the 26G needle (outer diameter 0.45 mm, inner diameter 0.26 mm), the 30G needle has a sharper tip, and its outer diameter is 50% smaller than that of the 26G needle. It passes through the eyeball wall more easily, with less damage, and has lower probability of uvea detachment and intraoperative bleeding. In the 15 patients in this study, a 30G needle with a length of 25 mm was used, and a nylon or prolene suture was loaded. The needle entered via limbal incision contralateral to the clefts and passed through the cyclodialysis cleft site with a continuous locking-stich suture pattern. During the operation, the tightness can be easily adjusted to observe whether the pupil was deformed.

However, since the inner diameter of the 30G needle is small, loading the nylon thread through the needle should be done under a microscope. Compared with the 26G needle, loading suture through the 30G needle is relatively time consuming. This suturing method is performed from the inside to the outside in the eye and is not suitable for patients with intact crystalline lens. In this study, all 15 patients were complicated with injured lens, which was removed during surgery. For severe ocular trauma, combined with PPV, this procedure has certain advantages and can reduce the number of scleral incisions to greatly reduce the operation difficulty and eliminate the long-term complications caused by scleral incisions. In the 15 patients, the cleft range did not exceed 180°. Considering that suture range exceed 180° is likely to lead to anterior segment ischaemia, we recommend secondary suture for a cleft over 180° after 1 month.

## Conclusions

In summary, continuous ab interno repairing of cyclodialysis cleft using a 30G needle in severe ocular trauma is a safe and effective procedure with simple operation, little tissue damage and few complications. It is a feasible technique if surgical indications are met.

## Data Availability

The datasets used and/or analysed during the current study available from the corresponding author on reasonable request.

## Abbreviations

IOP: Intraocular pressure
PPL: Pars plana lensectomy
PPV: Pars plana vitrectomy; phaco: phacoemusification
RD: Retinal detachment
UBM: Ultrasound biomicroscopy
VA: Visual acuity
